# Humanizing AI in medical training: ethical framework for responsible design

**DOI:** 10.3389/frai.2023.1189914

**Published:** 2023-05-16

**Authors:** Mohammed Tahri Sqalli, Begali Aslonov, Mukhammadjon Gafurov, Shokhrukhbek Nurmatov

**Affiliations:** ^1^Department of Economics, School of Foreign Services, Georgetown University in Qatar, Doha, Qatar; ^2^Department of Control and Computer Engineering, Politecnico di Torino, Turin, Italy; ^3^Department of Business Administration, Carnegie Mellon University in Qatar, Doha, Qatar

**Keywords:** human-AI interaction, Human-Computer Interaction, digital health, XAI, artificial intelligence

## Abstract

The increasing use of artificial intelligence (AI) in healthcare has brought about numerous ethical considerations that push for reflection. Humanizing AI in medical training is crucial to ensure that the design and deployment of its algorithms align with ethical principles and promote equitable healthcare outcomes for both medical practitioners trainees and patients. This perspective article provides an ethical framework for responsibly designing AI systems in medical training, drawing on our own past research in the fields of electrocardiogram interpretation training and e-health wearable devices. The article proposes five pillars of responsible design: transparency, fairness and justice, safety and wellbeing, accountability, and collaboration. The transparency pillar highlights the crucial role of maintaining the explainabilty of AI algorithms, while the fairness and justice pillar emphasizes on addressing biases in healthcare data and designing models that prioritize equitable medical training outcomes. The safety and wellbeing pillar however, emphasizes on the need to prioritize patient safety and wellbeing in AI model design whether it is for training or simulation purposes, and the accountability pillar calls for establishing clear lines of responsibility and liability for AI-derived decisions. Finally, the collaboration pillar emphasizes interdisciplinary collaboration among stakeholders, including physicians, data scientists, patients, and educators. The proposed framework thus provides a practical guide for designing and deploying AI in medicine generally, and in medical training specifically in a responsible and ethical manner.

## 1. Introduction

As the field of Artificial Intelligence (AI) is rapidly evolving, its applications on a plethora of domains are also expanding. One of those fields of application is medicine, and precisely the way medical training is delivered to practitioners. AI has the potential to revolutionize medical training by providing practitioners with advanced personalized tools and resources to improve their knowledge and skills. Thanks to machine learning and deep learning algorithms, medical training can be fully personalized and tailored to the trainee. Whether we take reading medical graphs like electrocardiograms (ECGs) (Tahri Sqalli et al., 2021a), or medical images like magnetic resonance images (MRIs) (Tahri Sqalli and Al-Thani, 2020), or even simulations like robotic surgery simulations (Poon et al., 2005; Tahri Sqalli et al., 2016; Yamashita et al., 2016), their repetitive aspect through human interpretation is a suitable process to generate patterns that can be processed by ML algorithms (Alahmadi and Saleem, 2022). However, incorporating AI into medical training raises important ethical considerations, including the need to ensure that AI systems align with human values and priorities (Bennett and Rosner, 2019). This is particularly important in the field of medicine, where the impact of AI can have far-reaching consequences for patients and practitioners alike. Humanizing AI in medical training is crucial for ensuring that AI systems align with the values, needs, and priorities of both practitioners and patients. This requires the consideration of factors such as the ethics of data collection and usage, the impact of AI on healthcare disparities, and the role of human oversight in AI systems.

Throughout our personal analysis of several technology-based medical tools and systems that incorporate AI, we realize that there are several different fields of medicine where humanizing AI in medical training is particularly important (Tahri Sqalli and Al-Thani, 2020). Among those fields is the cardiology field (Chetwood et al., 2012), where AI is increasingly being used to assist practitioners in interpreting electrocardiograms (ECGs). Another example is in the field of wearable devices for laypeople to monitor and improve their health conditions, where AI is being used to develop health coaching systems (Tahri Sqalli and Al-Thani, 2019) that can support individuals in managing their overall wellness.

This article aims to provide first-hand an overview of the considerations needed to humanize AI in medical training, drawing on examples from related fields like cardiology and wearable devices. We highlighting the key ethical considerations and best practices. This article also aims to inform the development of AI systems that are designed to support practitioners in providing high-quality care to their patients. This article targets mainly medical practitioners, medical students, AI engineers, researchers, computer scientists, designers, and patients who are either involved in the design or the use of those AI systems. The article proposes five pillars for responsible design: (1) transparency, (2) fairness and justice, (3) safety and wellbeing, (4) accountability, and finally (5) collaboration. The transparency pillar emphasizes the importance of ensuring that AI algorithms are explainable, while the fairness and justice pillar highlights the need to address biases in healthcare data and design models that prioritize equitable healthcare training outcomes. The safety and wellbeing pillar emphasizes designing AI models with patient safety and wellbeing as a top priority, while the accountability pillar calls for establishing clear lines of responsibility and liability for AI-derived decisions. Finally, the collaboration pillar calls for interdisciplinary collaboration to ensure that all stakeholders, including physicians, data scientists, and patients, are involved in the design and deployment of AI in medical training.

## 2. Responsible design pillars

### 2.1. Transparency pillar

As AI-enabled systems rapidly serve as mediators to consequential medical decision-making, their explainability is critical for end-users, whether these users are patients, medical trainees or practitioners. The systems' expalinability is important for taking informed and accountable actions. Studies assessing the process of explanation among human to human interactions found that it is most effective when the process is socially-situated (Ehsan et al., 2021). Moreover, AI systems are usually designed to be socio-organizationally embedded (Rezgui et al., 2005). Yet, Explainable AI (XAI) approaches have often adopted an algorithm-centered approach, focusing on the intricacies of the algorithm design, while forgetting the socially-situated aspect of the AI-enabled system's output (Rezgui et al., 2005). Thus, an imminent reform is needed toward developing socially-situated XAI. This would be through the introduction and exploration of Social Transparency (ST) within these AI-powered systems, especially in the context of medical training. To achieve this socio-technically oriented perspective, it is argued that while explainability is important for ensuring the trustworthiness of AI systems, it is not sufficient to address the broader concerns of social transparency (Rezgui et al., 2005). Thus, it is recommended that a framework for social transparency includes three components:

1. Descriptive transparency, which involves providing information about how the AI system works and how it was developed;
2. Procedural transparency, which involves making the decision-making processes of the AI system transparent and accountable; and
3. Substantive transparency, which involves ensuring that the outcomes of the AI system are aligned with societal values and goals.

This would expedite the infusion of the socio-organizational context into explaining AI-mediated decision-making system within the medical training context.

### 2.2. Fairness and justice pillar

Healthcare data is often biased due to the under-representation of certain populations in clinical trials and medical research. As it was demonstrated through ECG interpretation experiments (Tahri Sqalli et al., 2022b), racial and ethnic minorities are often underrepresented in healthcare research, which can lead to biased algorithms that fail to consider the unique health needs and outcomes of these populations (Fletcher et al., 2021). Similarly, gender biases in healthcare data can lead to disparities in diagnosis and treatment for women (Fletcher et al., 2021). Such biases in healthcare data can result in AI systems that are not representative of the diverse patient populations they serve and may even exacerbate health disparities. Moreover, AI models must be designed to prioritize equitable medical training outcomes. For instance, AI systems that are used in medical training should be designed to provide feedback and support that is tailored to the individual needs of trainees, regardless of their background or previous experience (Tahri Sqalli et al., 2022a). This helps mitigate the impact of biases in healthcare data and ensure that all trainees receive the support they need to succeed. Designing AI models that prioritize equitable medical training outcomes may help to address existing disparities in medical education and training. For example, individuals from disadvantaged backgrounds may face additional challenges in medical training due to their socio-economic status, lack of access to resources, and other factors. By designing AI models that account for these factors, healthcare educators can ensure that all trainees have an equal opportunity to succeed in their medical training (Tahri Sqalli and Al-Thani, 2020b).

### 2.3. Safety and wellbeing pillar

This pillar manifests as the most Human-centered pillar among the five pillars discussed. This is due to the severity of the threat that AI integration in medicare recommendations poses. Thus, the challenges and risks facing this process of integration. This in addition to challenges in policy and AI-based medicare recommendations. This pillar is thus based on the past technical failures that arose as a result of the early adoption of AI in hospitals. It also stems from our observations of the unavailability of the necessary polices of this integration. Moreover, our observations infer that medical practitioners lack experience in making AI-supported context-based decisions, mainly as a consequence of immature data strategies (Tahri Sqalli and Al-Thani, 2019).

The technological sophistication of AI software systems and their widespread applications in hospitals have increased risks of adverse events with patients ending up as victims (Makary and Daniel, 2016). Traditionally, if the patient has been the victim of a misdiagnosis, that has later resulted in the delay of the treatment plan, it was the physician who gets held responsible. With the introduction of AI, the main challenge that arises naturally is to develop a well-defined protocol of liability and involvement in case the patient is mistreated on the basis of an inaccurate analysis conducted by the AI (Makary and Daniel, 2016). Integration of artificial intelligence in modern hospitals and treatment practices introduces a significant level of automation in administrative work, hospital resource allocation, and treatment recommendations based on each patient's digital medical record. Automating these tasks enable medical professionals to focus on higher priority responsibilities that involve illness diagnosis, patient care, and making more efficient treatment plans. However, applications of AI in medicine and patient care require close observation and continuous improvement. This is to maximize the productivity of hospitals in operations and patient care. This would also ensure the utilization of AI software systems in medicare in a transparent, secure, and ethical manner (Makary and Daniel, 2016). Therefore, the practical steps that should be taken toward increasing the safety of AI systems and decreasing their errors in decision making procedures involve:

1. Testing: Continuous testing of the AI algorithms, and implementation of more robust data collection strategy.
2. Strong monitoring practices. This would be by setting in place data strategies, as well as best practices of the inclusion of AI within the hospital's operations.
3. Patient data privacy and protection, which is discussed thoroughly in the accountability pillar.

### 2.4. Accountability

In the medical field, clear lines separating the accountability of different stakeholders involved in the care for patients is critical. With the infusion of AI-based medical system supporting the provision of care to patients, accountability helps separate the decisions made by the AI from the ones made by medical practitioners (Feigenbaum et al., 2020). Accountability from the perspective of Human-Computer Interaction refers to the possibility of identification of a misbehaving actor whether a system or sub-system, the possibility for collecting evidence data and backlog in the case a system mis-behavior, and finally the availability of clear metrics for evaluation and judgement for whether the system's output is compliant with the medical's standard of care (Feigenbaum et al., 2020). Thus, it is recommended that a framework for accountability in the context of humanizing AI in medical training includes three components:

1. The ability to identify a misbehaving actor or subsystem (Feigenbaum et al., 2020). This is important as to find a clear separation between the decisions made by the human care provider as opposed to the recommendations provided by the supporting AI system. This in turn would help in fostering the application and the development of clearer care protocols, and thus effective incorporation of AI-powered systems in the delivery of care.
2. The ability to identify root causes behind an AI system's decision or output (Feigenbaum et al., 2020). Another method to ensure accountability is through tracing back to the root causes. This method manifests in the medical practice. When the standard of care is violated through a non-compliant system's output, it can be noticed and caught prior and while in operation by the supervising medical practitioner. At the same time, the misbehaving sub-system is found by reviewing the back-log data.
3. The possibility of Acquisition, preservation, and use of evidence (Feigenbaum et al., 2020). This refers to the AI sub-systems having enough documented processes and retrievable raw data needed to assess its overall performance. This will support in the delivery of strong evidence for the parties accordingly.

### 2.5. Collaboration

We refer to the The term “collaboration” in the field of healthcare when medical professionals take roles and share responsibilities to solve problems and make decisions to improve patients' health. With the wide use of AI in healthcare, collaboration of different parties have become significant, specifically between healthcare educators, technology experts and other stakeholders. Lomis et al. (2021) pointed out various areas which highlight the significance of collaboration. Mainly for the development of AI tools and platforms, for health monitoring, for data infrastructure management, and for evaluation and assessment. Healthcare providers and educators should be prepared for changes in inter-professional practice trends. The notion of a health care provider will change from being a human who manages information to being a systems-thinking professional who can access, evaluate, and use information to meet the requirements of a specific person or community (Lomis et al., 2021). Each practitioner must learn how data inputs affect AI outputs that directly impact patients' care and therapy. AI-connected wearables will increasingly combine the delivery of treatment and prevention (Tahri Sqalli and Al-Thani, 2020a). Thus, training in digital health literacy is required. This is to help healthcare professionals understand such data through the perspective of a particular health profession, as well as to understand how such information fits within the domain of other health professions and the larger healthcare system (Tahri Sqalli and Al-Thani, 2020a). Data collected by patients themselves through smart technology monitoring will supplement data submitted by each care provider. Students studying for careers in the health professions need to be prepared for the paradigm change that is moving away from “disease-oriented medicine” toward wellness promotion in the framework of a partnership with patients and providers (Lomis et al., 2021). When healthcare clinicians work with data scientists and other digital expertise, the inter-professional team's composition will change. The team's responsibilities will change to a more interdisciplinary, person-centered approach, directing patients toward healthier options and deep learning-based decision-making. As teams increasingly rely on data-driven algorithms to guide decision making, educators should develop inter-professional learning opportunities to identify data biases, bioethical difficulties, and risks for liability.

## 3. Discussion

The proposed responsible design pillars show that the process of humanizing AI in medical training is an interdisciplinary and collaborative process. It requires a collaborative effort between patients, medical practitioners, AI developers, policymakers, and other stakeholders. Thus, the need for the adoption of a “human-centered” approach to AI development. This approach involves taking into consideration the social, ethical, and legal implications of their work. Policymakers, on the other hand, should create regulations and standards that promote social transparency in AI systems. Due to the interdisciplinary nature of the task, several challenges arise. These challenges may be medical, technical, or policy related. Throughout the discussion we briefly shed light on these challenges and explain how the proposed pillars may be an asset to overcoming them.

Medical error is considered one of the most important adverse events affecting the medical sector. This is more consequential with respect to patients whose standard of care includes an involvement of AI in the decision making process. In the analysis carried out by Makary and Daniel (2016), the mean rate of death from medical errors across the US is found to reach an average of 251,454 per year across the total number of US hospital admissions. Moreover, the CDC rankings suggest that medical error is the third most common cause of death in the US (Makary and Daniel, 2016). Although the computing power of AI to dwell on large sets of patient digital data has benefits to identify early-stage illnesses or patterns that cannot be immediately seen by medical professionals, effective data strategy is of critical value to ensure that the recommendations produced by AI systems are less prone to medical errors. Indeed, data collection has to be as broadly inclusive as possible, taking into consideration age, race, and gender of patients, and reducing the bias toward under-represented patient groups, whose data might not have been taken into account in earlier AI integration stages (Tahri Sqalli et al., 2021b).

Expanding on the risks of medical error, another challenge to these systems is the technical challenge. Overcoming this technical challenge would be through the increase of the safety of AI systems in medicare. It would be effectively done by continuously auditing AI systems operating within hospitals. It would also be a necessity to closely monitor any edge cases with regards to individual patients and see how AI came to the error analysis in the specific instances. This is mainly due to the fact that even machine learning algorithms that produce data-driven analytics can be wrong. An example of that would be when an AI system fails to identify a tumor on the scan, or when it recommends the wrong treatment plan including the improper drug. These repercussion have a direct impact on the patient who is the end user of the system to be affected (Price, 2022). The issue of injury caused by the wrong recommendation of the AI system can even exacerbate with the standardization of the system across the whole hospital. The error of the AI system can translate from a limited group of individual patients to more (Price, 2022).

The last challenge to be overcome is the achievement of social transparency in AI systems. This is mainly due to the lack of standardization and consistency in how AI systems are developed, evaluated, and deployed. Developing standards and best practices for AI-based medical training systems could help promote social transparency and ensure that these systems are trustworthy and reliable. This will eventually overcome the complexity and opacity of the input and parameters of these AI systems, and hence facilitate the understanding of how they operate and how decisions are made. Moreover, developing tools and methods for explaining the function of AI systems to non-experts could help address this challenge and improve social transparency.

**Figure 1:**
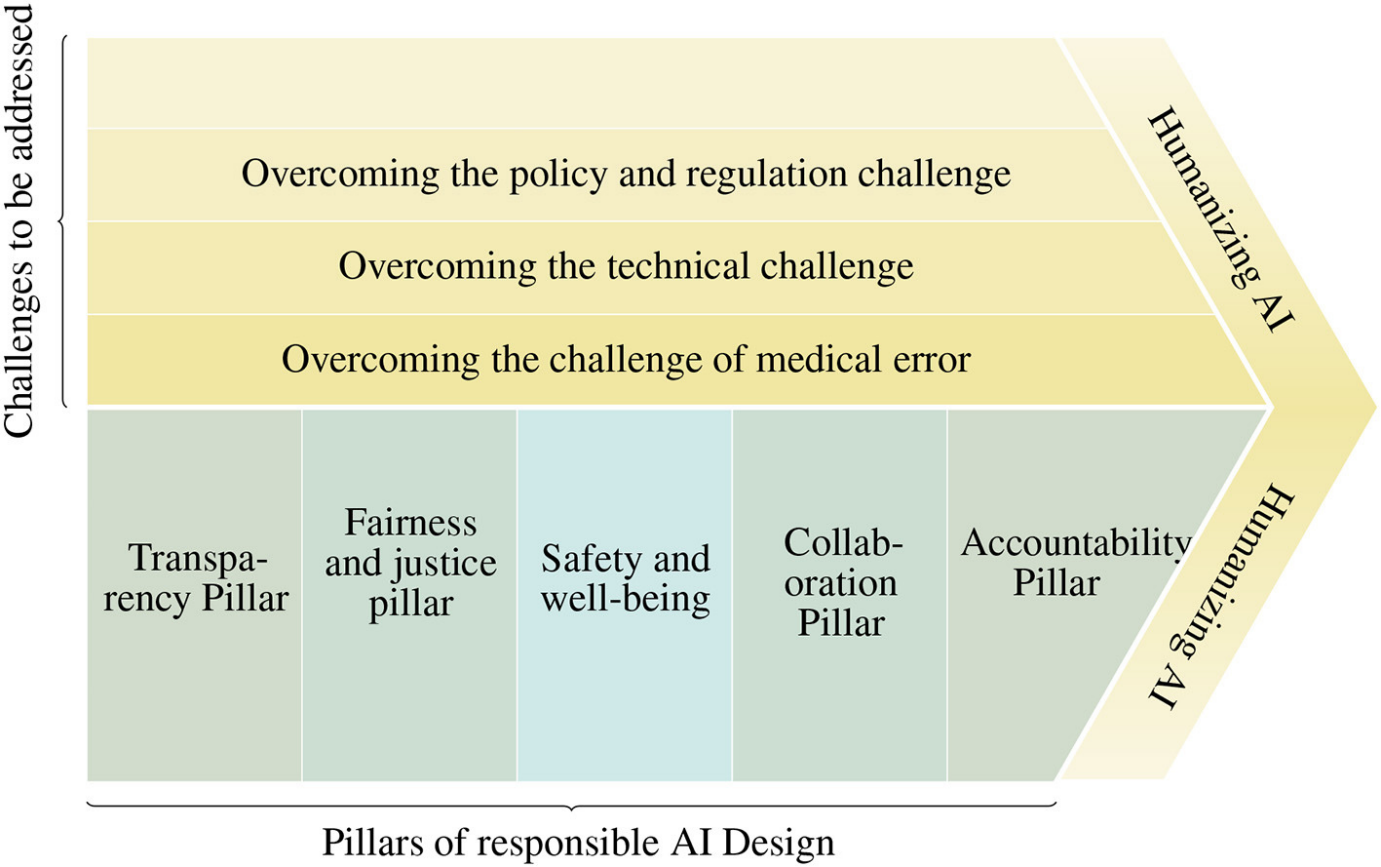
Ethical framework for responsibly designing medical training systems.

Figure 1 summarizes the proposed framework including the pillars and the challenges that need to be overcome for the responsible design of medical training systems.

## 4. Conclusion

In conclusion, the increasing use of AI in healthcare necessitates the consideration of ethical principles in the design and deployment of its algorithms, particularly in medical training. This perspective article provides a framework for responsible AI design, drawing on past research in electrocardiogram interpretation training and e-health wearable devices. The proposed framework comprises five pillars: transparency, fairness and justice, safety and wellbeing, accountability, and collaboration. These pillars emphasize the importance of maintaining the explainability of AI algorithms, addressing biases in healthcare data, prioritizing patient safety and wellbeing, establishing clear lines of responsibility and liability for AI-derived decisions, and fostering interdisciplinary collaboration among stakeholders. By adopting this framework, the design and deployment of AI systems in medical training can promote equitable healthcare outcomes for both medical practitioners trainees and patients while ensuring adherence to ethical principles.

## Data availability statement

The original contributions presented in the study are included in the article/supplementary material, further inquiries can be directed to the corresponding author.

## Author contributions

MT contributed the conception and brainstorming of the article, organized the structure of the article, and wrote the manuscript. BA, MG, and SN have contributed with writing one pillar among the proposed 5 pillars. All authors contributed to manuscript revision, read, and approved the submitted version.
